# Articular Cartilage Repair Using Marrow Stimulation Augmented with a Viable Chondral Allograft: 9-Month Postoperative Histological Evaluation

**DOI:** 10.1155/2015/617365

**Published:** 2015-01-01

**Authors:** James K. Hoffman, Sandra Geraghty, Nicole M. Protzman

**Affiliations:** ^1^Department of Orthopedics, Coordinated Health, 2775 Schoenersville Road, Bethlehem, PA 18017, USA; ^2^Product Development Department, Osiris Therapeutics, 7015 Einstein Drive, Columbia, MD 21046, USA; ^3^Clinical Education and Research Department, Coordinated Health, 3435 Winchester Road, Allentown, PA 18104, USA

## Abstract

Marrow stimulation is frequently employed to treat focal chondral defects of the knee. However, marrow stimulation typically results in fibrocartilage repair tissue rather than healthy hyaline cartilage, which, over time, predisposes the repair to failure. Recently, a cryopreserved viable chondral allograft was developed to augment marrow stimulation. The chondral allograft is comprised of native viable chondrocytes, chondrogenic growth factors, and extracellular matrix proteins within the superficial, transitional, and radial zones of hyaline cartilage. Therefore, host mesenchymal stem cells that infiltrate the graft from the underlying bone marrow following marrow stimulation are provided with the optimal microenvironment to undergo chondrogenesis. The present report describes treatment of a trochlear defect with marrow stimulation augmented with this novel chondral allograft, along with nine month postoperative histological results. At nine months, the patient demonstrated complete resolution of pain and improvement in function, and the repair tissue consisted of 85% hyaline cartilage. For comparison, a biopsy obtained from a patient 8.2 months after treatment with marrow stimulation alone contained only 5% hyaline cartilage. These outcomes suggest that augmenting marrow stimulation with the viable chondral allograft can eliminate pain and improve outcomes, compared with marrow stimulation alone.

## 1. Introduction 

Articular cartilage injuries of the knee are fairly common, affecting an estimated 900,000 Americans each year [1–4]. Clinically, these chondral defects result in pain, swelling, disability and, with continued cartilage deterioration, osteoarthritis. Unfortunately, articular cartilage has a limited intrinsic repair capacity, primarily due to its avascular nature [5–7].

The most common reparative procedures for articular cartilage defects of the knee include marrow stimulation, osteochondral allografts, osteochondral autografts, and autologous chondrocyte implantation. Developed by Steadman in the 1980s, marrow stimulation is the most frequently performed reparative procedure [8–10].

Marrow stimulation is generally employed as the first line of treatment. It preserves the integrity of the knee, which, if marrow stimulation fails, allows for subsequent more invasive revision procedures to be performed. With standard marrow stimulation techniques, damaged cartilage is debrided, the calcified cartilage layer is removed, and the subchondral bone plate is uniformly penetrated, enabling blood and bone marrow containing mesenchymal stem cells (MSCs) to enter the defect space. By accessing the underlying bone marrow, a biologic repair response is initiated. Marrow stimulation is most successful when used in younger patients (<40 years old) with small (<2 cm^2^), isolated chondral defects [11–14]. However, the long term success of marrow stimulation is limited, especially in older patients with large defects [15–17]. Typically, marrow stimulation repair results in fibrocartilage formation within the defect space. Fibrocartilage is primarily comprised of type I collagen as opposed to type II collagen, which is found in healthy hyaline articular cartilage [15–17]. Due to its altered composition, fibrocartilage does not have the same biomechanical properties as native articular cartilage and cannot withstand the normal physical stresses endured by articular joints [5]. This leads to long term problems including poor biomechanical performance, abnormal bone growth, and an increased risk of developing osteoarthritis [18, 19]. Deterioration of patient outcomes following marrow stimulation often necessitates further, more invasive interventions [20].

Several techniques have been proposed to augment marrow stimulation with the goal of directing repair toward hyaline cartilage rather than fibrocartilage. Ultimately, restoration of hyaline cartilage will achieve better long term outcomes following marrow stimulation surgery. Scaffolds are theorized to secure the blood clot throughout the defect space, providing structural support and facilitating cell adhesion and migration during the repair process. Polyglycolic acid, chitosan-glycerol phosphate, chondroitin sulfate, polyethylene glycol, xenogeneic collagen (types I and III), and micronized allogeneic cartilage have all been studied as scaffolds for marrow stimulation augmentation [21–28]. Though predominantly limited to animal studies and case reports, preliminary data suggest that augmentation of marrow stimulation with scaffolds may encourage the formation of more hyaline-like repair tissue that demonstrates a more organized architecture. Scaffolds can also serve as a delivery vehicle for cultured cells and/or growth factors. Dorotka et al. found more hyaline-like repair tissue in a sheep model when a collagen scaffold was seeded with cultured autologous chondrocytes and used to augment marrow stimulation [29, 30]. Other researchers have proposed using hyaluronic acid and various growth factors to augment marrow stimulation with the goal of promoting MSC proliferation and differentiation [31–35]. Cytokine inhibitors have also been employed to block inflammatory cytokines and decrease proteoglycan breakdown in repair tissue [36].

Recently, a cryopreserved viable chondral allograft (Cartiform, Osiris Therapeutics, Inc., Columbia, Maryland) was developed to augment marrow stimulation. The chondral allograft contains viable cells, extracellular matrix proteins, and chondrogenic factors in the 3-dimensional architecture of healthy articular cartilage. With the addition of pores, the chondral allograft was designed to enable chondrocyte cryopreservation, increase flexibility, and facilitate MSC infiltration upon implantation. By combining several promising augmentation techniques (scaffold, cells, and growth factors) in a single augmentation strategy, the chondral allograft creates the optimal microenvironment for repair. Moreover, the chondral allograft can be easily cut to the desired shape, conformed to contours of all chondral surfaces, and fixed in place with fibrin glue and/or sutures in a simple, single-stage procedure. With a shelf life of two years when stored between −75°C and −85°C, the chondral allograft is also available on demand. The present report describes treatment of a trochlear defect with marrow stimulation augmented with this novel cryopreserved chondral allograft. The repair tissue histology is evaluated at nine months and compared to repair tissue histology following treatment with marrow stimulation alone.

## 2. Case Presentation

### 2.1. Clinical History

**Figure 1:**
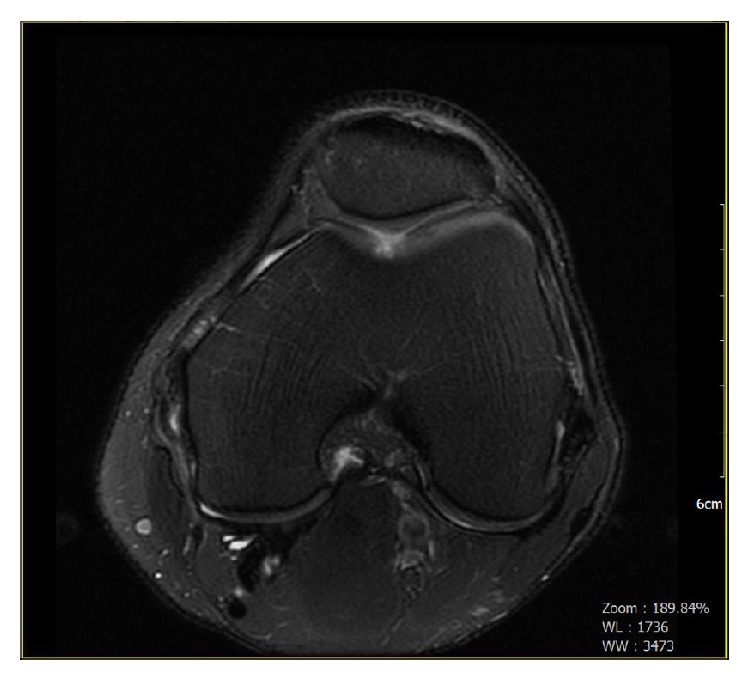
Preoperative radiograph. Multiplanar, multisequential images were obtained through the left knee on a 1.5 Tesla MRI scanner. Mild patellofemoral joint osteoarthritis. A focal region of grades III-IV chondromalacia overlying the central femoral trochlear groove.

In December 2012, a 50-year-old man presented with ongoing medial left knee pain. He reported a recent valgus injury to his knee while playing ice hockey. His surgical history indicated a previous meniscal surgery on the left knee. Magnetic resonance imaging (MRI) demonstrated a sprain of the medial collateral ligament at the proximal femoral attachment. Mild patellofemoral joint osteoarthritis was noted with a focal region of grade three to grade four chondromalacia overlying the central femoral trochlear groove. Additionally, there was chondral fraying and small chondral fissures were overlying the far lateral aspect of the lateral patellar facet (Figure 1).

Conservative treatment failed to improve the clinical symptoms. Surgical management was discussed with the patient and the risks and benefits were reviewed. The patient elected to undergo arthroscopy of the left knee. In January 2013, an arthroscopy of the knee revealed a 5 mm by 10 mm trochlear defect. Considering the size of the defect and the fact that the rest of the knee joint was in good condition, the surgeon proceeded with marrow stimulation augmented with the chondral allograft.

### 2.2. Operative Intervention for Cartilage Repair

**Figure 2:**
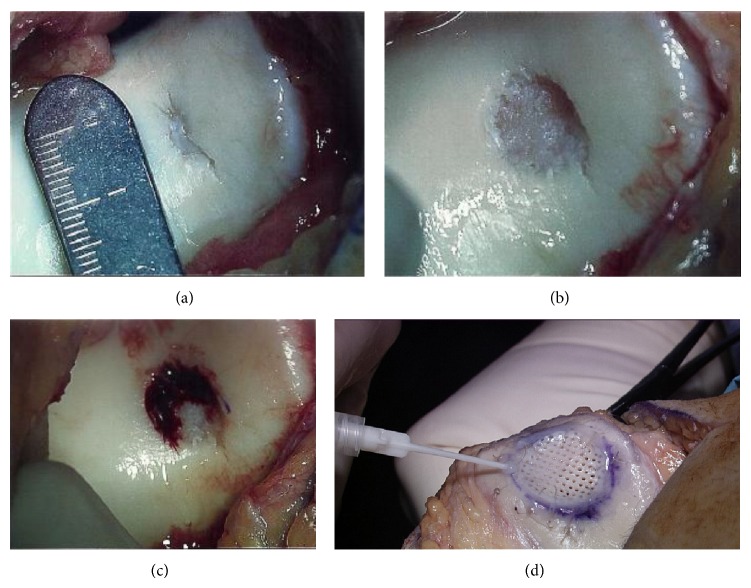
(a) Defect. The defect was inspected intraoperatively. The fissure in the trochlea measured approximately 5 mm in diameter and 10 mm in length. (b) Removal of damaged cartilage. Loose fragmenting cartilage was removed until healthy, viable cartilage was encountered. The defect space then measured approximately 10 mm by 10 mm. (c) Marrow stimulation. The base of the defect was drilled to facilitate blood flow from the bone marrow into the defect space. (d) Chondral allograft application. This is a representative photo from a different case, demonstrating the final application of the chondral allograft. Note that the free edges of the chondral allograft were sutured to surrounding cartilage and a fibrin sealant was then applied to stabilize the chondral allograft within the lesion site.

An anterior incision was used. A medial parapatellar incision was made, and the patella was lateralized, providing visualization of the trochlear defect (Figure 2(a)). The knee was placed in flexion on the operating room table. The edges of the trochlear defect were debrided by curettage and sharp dissection. After loose fragmenting cartilage was removed, the defect measured 10 mm by 10 mm (Figure 2(b)). Marrow stimulation was performed using 0.045 Kirschner wires to drill the base of the defect and facilitate bleeding from the bone marrow (Figure 2(c)). The chondral allograft (Cartiform, Osiris Therapeutics, Inc., Columbia, MD) was cut in size from 20 mm in diameter to 10 mm in diameter to fit the defect. The edges of the chondral allograft were secured to the surrounding healthy cartilage using #4.0 Vicryl sutures. A fibrin sealant (Tisseel, Baxter, Deerfield, IL) was then applied to the periphery of the allograft to further stabilize it within the chondral defect (Figure 2(d)). The fibrin glue is used for initial stabilization, and it is imperative that the docking sites for the bone marrow MSCs are uncongested and available. The patella was then carefully repositioned over the graft with the knee in extension. The lower extremity was placed in a total range of movement immobilizer with the limb extended. The patient was provided instructions for utilization of aspirin for deep venous thrombosis prophylaxis.

### 2.3. Postoperative Rehabilitation

Postoperatively, the patient was initiated on foot-flat, crutch ambulation. At the three-week visit, the patient was permitted to unlock the brace to 90° to allow for gravity assisted range of motion. Isometric exercises were initiated and electrical stimulation was performed to the quadriceps. From postoperative week six to postoperative week twelve, full range of motion was established. A postoperative MRI was obtained at 12 weeks, which verified that the trochlear defect was filled with the chondral allograft. Strengthening exercises were also initiated at 12 weeks. Five months following surgery, the patient reported no pain and demonstrated restoration of strength and functionality of the left knee. He reported the ability to perform all activities of daily living without complication. His knee demonstrated full range of motion. At the nine-month follow-up, the patient had returned to sport, participating in both hockey and softball. However, he reported pain and weakness of the shoulder secondary to falling on an outstretched arm approximately seven months after the cartilage repair. Follow-up MRIs revealed a rotator cuff tear in the left shoulder and a medial meniscal tear in the left knee. The patient consented to a rotator cuff repair, a partial medial meniscectomy, and a biopsy of the previous cartilage repair site. An MRI was ordered to examine the trochlear repair, and the procedure was scheduled for October 2013, nine months following the repair of the trochlear defect.

### 2.4. Imaging

**Figure 3:**
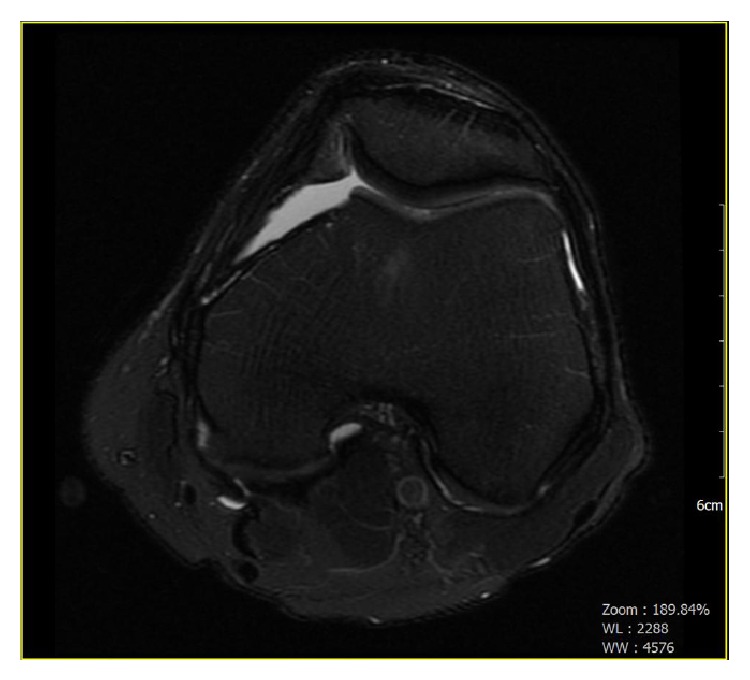
Nine-month postoperative radiograph. Unenhanced MRI of the left knee performed on a 1.5 Tesla magnet. Examination of the area of the femoral patellar joint where the patient is status-post a chondroplasty of the trochlear groove shows low T1 and high T2 signals in the subchondral bone. The articular surface of the chondroplasty defect shows a signal, which is isointense with articular cartilage. The thickness of the signal is less than that of the adjacent cartilage.

An unenhanced MRI of the left knee was performed on a 1.5 Tesla magnet, nine months following repair with a chondral allograft (Figure 3). Examination of the trochlear groove showed low T1 and high T2 signals in the subchondral bone. Compared with the prior exam at 12 weeks, the corresponding signal was relatively higher on the T1 portion of the study. The articular surface of the chondroplasty defect showed a signal, which was isointense with articular cartilage.

### 2.5. Partial Meniscectomy and Cartilage Biopsy from Trochlear Repair

**Figure 4:**
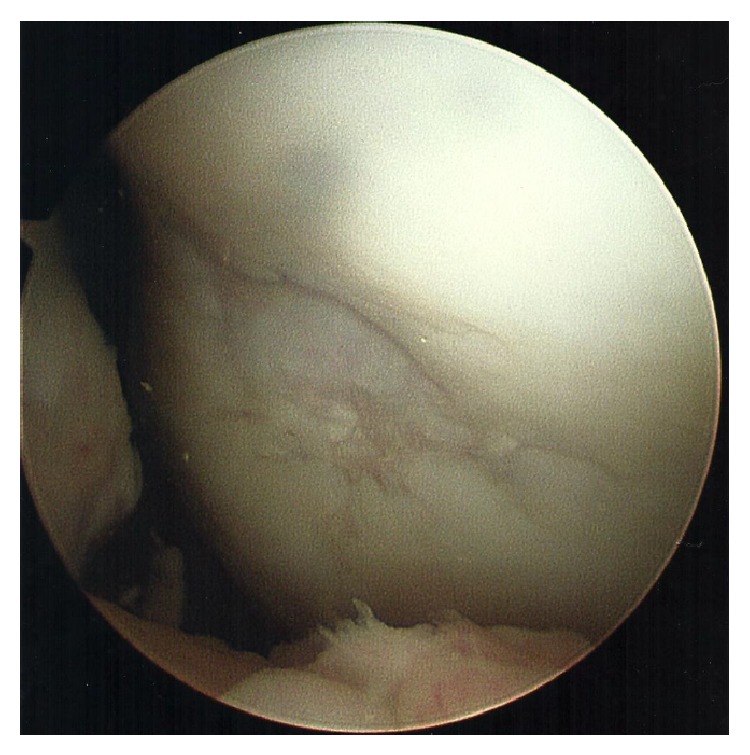
Repaired trochlear defect. Nine months following the trochlear defect repair, the repaired trochlear was visualized. Of note, there was a hyaline-like material and fraying of hyaline cartilage around the damaged area. No bare bone was observed. The fraying around the graft was determined to be clinically inconsequential and did not represent interference of stable fixation between the underlying graft and the bone. The density of the repair tissue was consistent with the surrounding, normal chondral tissue.

An inferomedial portal and an inferolateral portal were made. A double-port arthroscope was introduced into the inferolateral portal. Attention was directed to the region of the previously repaired trochlea. A material that appeared to be hyaline cartilage covered the lesion. Fraying of cartilage around the lesion was observed, but no bare bone was noted (Figure 4). The fraying was found to be clinically inconsequential and did not present interference of stable fixation between the underlying graft and the bone. The density of the repair tissue was consistent with the surrounding, normal chondral tissue. The hyaline cartilage on both femoral condyles was intact. A partial medial meniscectomy was performed. A central portal site was made, a 1.5 mm punch biopsy was advanced, and a sample of the repair tissue along with approximately two millimeters of underlying bone was obtained from the trochlea. The cartilage in the trochlear region was probed and found to be stable and connected to the underlying bone.

### 2.6. Additional Biopsy Samples

Two additional biopsies were collected for comparison purposes. A biopsy was obtained from a 43-year-old patient 8.2 months after a failed marrow stimulation surgery. This marrow stimulation repair tissue did not maintain its structure upon removal from the patient, but rather several pieces of tissue were recovered and analyzed. A biopsy of healthy articular cartilage and underlying bone was obtained from the trochlea of a cadaver as well to serve as a positive control. This biopsy was collected using the same type of 1.5 mm diameter biopsy punch that was used to collect the chondral allograft repair tissue biopsy.

### 2.7. Histology

All samples were fixed in 4% paraformaldehyde, decalcified in 10% formic acid, processed in paraffin, and embedded. Five-micron-thick longitudinal sections were obtained down the center of the biopsies. The sections were stained with hematoxylin and eosin (H&E) and safranin O (SAFO). Additionally, immunohistochemistry was performed on sections using antibodies against type I and type II collagen. To perform this analysis, sections were deparaffinized and rehydrated in distilled water, and endogenous peroxidase activity was quenched with 3% hydrogen peroxide. The sections were next treated with proteinase K for type I collagen staining or chondroitinase for type II collagen staining and blocked with serum-free protein block (DAKO, Carpinteria, California, USA). The sections were then treated with rabbit polyclonal type I collagen (AbD Serotec, Raleigh, North Carolina, USA) at 1 : 200 for 30 minutes or rabbit polyclonal type II collagen (MD Biosciences, St. Paul, Minnesota, USA) at 1 : 200 for 60 minutes. The sections were treated with a polymer detection system (DAKO Rabbit Envision+ HRP) for 30 minutes. The reaction product was detected with 3,3′-diaminobenzidine (DAB) and sections were counterstained in Mayer hematoxylin (DAKO). In the negative control sections, normal rabbit serum was substituted for the primary antibody at the same protein concentration as the primary antibody. Positive controls consisted of sections of normal human subchondral bone (for type I collagen) and articular cartilage (for type II collagen). All samples were analyzed by an experienced, board-certified veterinary pathologist. Each section was carefully evaluated and graded according to previously published methods of the International Cartilage Repair Society (ICRS) II scoring system [37].

### 2.8. Clinical and MRI Results

The MRI obtained 12 weeks following treatment with marrow stimulation augmented with a viable chondral allograft showed that the chondral allograft remained in place, filling the defect. Five months following the intervention, the patient reported no pain, demonstrated full range of motion, and could perform all activities of daily living. Nine months following the intervention, the patient had returned to sport and an MRI revealed that the repair tissue was isointense with the surrounding articular cartilage. Also at nine months, arthroscopic examination of the cartilage repair tissue revealed that it was stable and nonfriable upon probing. The lesion had filled and demonstrated osseous incorporation and surface congruency. The repair tissue was well fixated to the underlying bone and surrounding cartilage.

### 2.9. Histology Results

**Figure 5:**
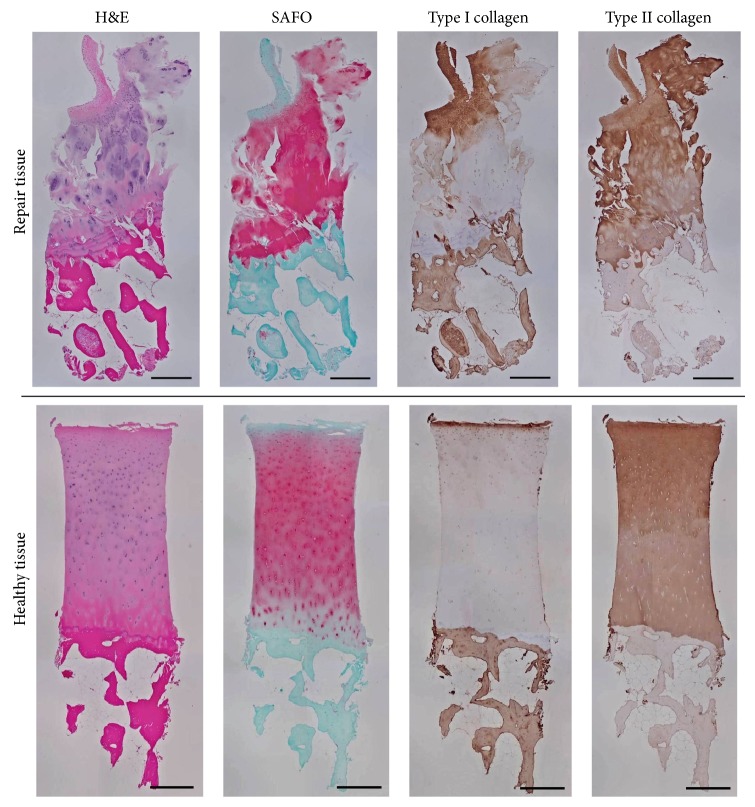
Histological stains. Histological stains of the biopsies of cartilage and underlying bone that were taken from the site of the chondral allograft implantation on the trochlea at nine months following the repair (repair tissue) and from the trochlea of a cadaver (healthy tissue, for comparison). Scale bars = 500 *µ*m.

**Figure 6:**
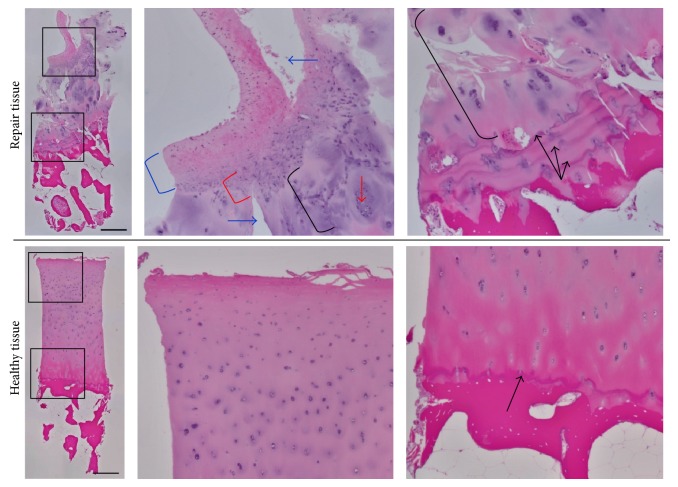
Hematoxylin and eosin stain. Hematoxylin and eosin stain of patient repair tissue and healthy tissue with higher magnification of the superficial and deep zones of the articular cartilage. The superficial zone of the repair tissue contains a layer of fibrocartilage (blue bracket) that transitions to hyaline-like cartilage (red bracket) and then hyaline cartilage (black bracket), with some degenerative changes including chondrocyte clustering (red arrow) and fissuring (blue arrows). The deep zone contains hyaline cartilage (black bracket) with a replicated tidemark (black arrows). The healthy tissue is entirely hyaline cartilage with an established tidemark (black arrow) between the cartilage and the bone. Scale bars = 500 *µ*m.

Table 1 outlines the ICRS II histological scores for the three biopsies that were analyzed. The articular cartilage that was biopsied from a cadaver was determined to be 100% hyaline cartilage and serves as an example of healthy articular cartilage for comparison (Figures 5-6). The biopsy collected nine months following marrow stimulation augmented with a viable chondral allograft contained abundant type II collagen with type I collagen only present in the superficial zone (Figure 5). The presence of type II collagen is indicative of tissue resembling hyaline cartilage and contributed to the analysis that the biopsy was predominantly hyaline cartilage repair tissue (85%) (Table 1). As shown in Figure 6 and outlined in Table 1, the biopsy also contained hyaline-like cartilage (5%), fibrocartilage (5%), and degenerative tissue (5%), primarily in the superficial zone with some chondrocyte clustering and fissuring. Safranin O staining revealed ample proteoglycan content throughout the majority of the biopsy (Figure 5). A closer evaluation of the deep zone of this biopsy revealed a replicated tidemark, indicating rapid advance of the mineralization front and strong integration between the repair tissue and the underlying bone (Figure 6). Additionally, the biopsy revealed no signs of inflammation, abnormal calcification/ossification, or vascularization (Table 1).

**Figure 7:**
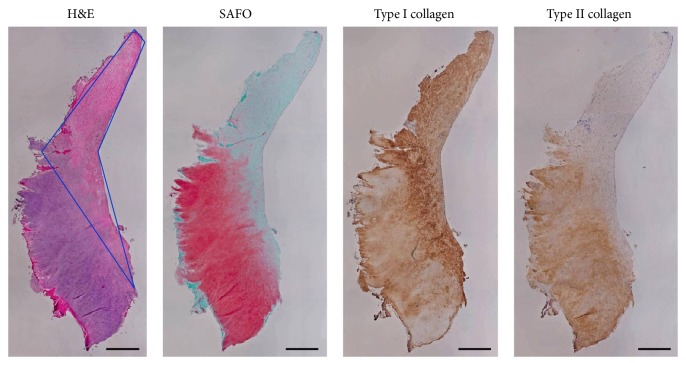
Histological stains after marrow stimulation. Histological stains of cartilage repair tissue collected from 43-year-old patient 8.2 months after marrow stimulation surgery that required revision. Section outlined in blue in H&E stain is fibrocartilage, while the remaining tissue is primarily hyaline-like cartilage. Scale bars = 500 *µ*m.

The biopsy that was collected from another patient 8.2 months following a marrow stimulation procedure without implantation of a chondral allograft lost its structure and was not attached to bone, but it was analyzed as fully as possible. As outlined in Table 1, this biopsy contained 45% hyaline-like cartilage, 40% fibrocartilage, 5% hyaline cartilage, 5% fibrous connective tissue, and 5% degenerative tissue. The biopsy contained more type I collagen than type II collagen and proteoglycans were found predominately in the hyaline-like cartilage tissue segment (Figure 7). Cell morphology was worse in this biopsy than the chondral allograft biopsy. Additionally, areas of vascularization were visible in the biopsy from the patient treated with marrow stimulation alone.

## 3. Discussion

This is the first report to evaluate repair tissue following the use of a viable chondral allograft to augment marrow stimulation for articular cartilage repair. This novel augmentation strategy consists of allograft cartilage that has been penetrated with pores. The pores increase flexibility, facilitate MSC infiltration, and enable chondrocyte cryopreservation. When the chondral allograft is needed, it can be quickly thawed, while maintaining all of the contents of fresh healthy cartilage, namely, viable chondrocytes, extracellular matrix proteins, and chondrogenic growth factors. The chondral allograft is most similar to a fresh osteochondral allograft, minus the presence of bone. As such, the chondral allograft builds on the 40+ years of safety and efficacy research describing osteochondral allografts [38, 39]. Unlike the implantation of osteochondral allografts, implantation of the chondral allograft does not require size or contour matching, as the graft can be cut to fit the size of the defect. Penetration of the subchondral bone with marrow stimulation enables MSCs to enter the defect space where they infiltrate and attach to the three-dimensional scaffold within the pores of the chondral allograft. The active chondrocytes within the chondral allograft secrete chondrogenic growth factors, which direct the MSC population to undergo chondrogenesis. Over time, the implanted chondral allograft is expected to become integrated with the host cartilage and to remodel and function as mature, healthy cartilage.

Following a marrow stimulation procedure, the best results reportedly occur when the initial lesion completely fills with a stable blood clot and the patient adheres to the recommended postoperative regimen [40, 41]. Additionally, smaller lesions (<2 cm^2^) and younger patients (<40) have been shown to demonstrate superior outcomes [14, 42]. Even so, with traditional marrow stimulation, MSCs typically differentiate into fibrochondrocytes and produce fibrocartilage tissue. Evidence suggests that the fibrocartilage repair tissue has a limited lifespan and physiologic competence [13, 43]. Despite its shortcomings, marrow stimulation remains popular and outcomes have been well documented over the past 20 years.

In a review of 29 marrow stimulation cases in young patients (average age: 24.3 years), Gudas et al. found that only 49% had good-to-excellent results twelve months after surgery [44]. These repair evaluations were based on the lesion fill, osseous incorporation, and surface congruency evidenced radiographically [44]. Nine of the 29 patients required revision surgeries due to loosening of the fibrocartilage repair tissue and arthrofibrosis. In 14 of the 29 patients, biopsies were collected and analyzed histologically. Fibrocartilage was found in 57% of the biopsies while the other 43% consisted of soft fibroelastic repair tissue. These findings suggest that, even in the ideal subset of patients, the results following standard marrow stimulation are less than ideal.

In another study, biopsies were obtained twelve months after marrow stimulation in 38 patients [45]. The biopsies were evaluated using a visual analog scale from 0 to 100 for several parameters [45]. Average scores ranged between 10 and 65 for matrix staining, cell morphology, chondrocyte clustering, and surface architecture [45]. These histological scores were similar to the scores for the marrow stimulation alone biopsy in the present report, which demonstrated 45% hyaline-like cartilage/40% fibrocartilage and received similar specific parameter scores. Because this marrow stimulation biopsy was collected at approximately the same postsurgery time point as the chondral allograft biopsy and was from a patient of a similar age (marrow stimulation: 8.2 months from 43 years old, chondral allograft: 9 months from 50 years old), it served as a suitable comparison.

The MRI of the patient's repair tissue nine months following marrow stimulation augmented with the chondral allograft revealed that the defect was filled, congruent with the surrounding tissue, and incorporated into the existing osseous framework. Upon arthroscopic probing of the chondral allograft repair tissue, it was determined that the repair tissue was well adhered to the underlying bone and resembled hyaline cartilage in its firmness and appearance. In comparison, traditional marrow stimulation procedures occasionally result in tissue that loosens from the underlying bone [44]. Fibrocartilage is also more compliant when mechanically probed.

Functionally, the patient who received the chondral allograft was able to perform all activities of daily living without complication five months after surgery and returned to full participation in sport nine months after surgery. In comparison, Gudas et al. found that only 52% of patients treated with unaugmented marrow stimulation were able to return to sport at their preinjury level after 6.5 months [44]. Even professional athletes who strictly abided by the rehabilitation protocol following marrow stimulation have been found to take 7.5 months to return to play and 21% are unable to return at all [46]. The histological analysis of the biopsy that was collected from the patient who received the chondral allograft revealed that the repair tissue was comprised primarily of hyaline cartilage with a thin layer of fibrocartilage in the superficial zone. The strong staining for type II collagen and aggrecan within the biopsy suggests that the repair tissue had the tensile strength and load absorption capabilities required to withstand the forces exerted on articular cartilage. Additionally, cells were distributed throughout the biopsy, there was excellent basal integration, and there were no significant abnormalities in either the cartilage or the underlying bone. Notably, hyaline repair tissue was found in 85% of the biopsy following marrow stimulation augmented with the chondral allograft, compared with only 5% of the biopsy following marrow stimulation alone. These outcomes suggest that augmenting marrow stimulation with the viable chondral allograft does, in fact, result in superior outcomes than marrow stimulation alone.

There is only one other product (BST-CarGel, Piramal Life Sciences, Laval, Quebec) designed to augment marrow stimulation that has been histologically evaluated through biopsies collected from clinical cases. The product is a chitosan-glycerol phosphate scaffold used to solidify the clot following marrow stimulation. Biopsies have revealed better ICRS II histology scores for the augmented group compared with the marrow stimulation group (overall scores of 64.5 versus 36.9) [47]. Presently, this product is not available for use in the United States. Most other outcome data on marrow stimulation augmentation strategies are limited to in vitro studies and in vivo animal models [21]. To date, no other marrow stimulation augmentation technique supplies the combination of viable chondrocytes, extracellular matrix proteins, and growth factors within the lesion space. While a single biopsy may not translate to all patients or be representative of the entire region of repaired cartilage, its analysis and comparison with other biopsies provide insight into the physiologic outcomes following marrow stimulation augmented with this viable chondral allograft. A multicenter study is presently underway to review the results of a greater patient population to fully understand and characterize outcomes following this treatment.

The results from this initial report demonstrate that this technique, employing allograft cartilage to facilitate repair, results in better repair tissue with features resembling hyaline cartilage than marrow stimulation alone. In the patient studied, this procedure effectively afforded full restoration of function and resolution of pain. The authors believe that this technique offers a means of improving the previously utilized marrow stimulation procedures and will allow athletes and patients with chondral defects in otherwise healthy knees to return to normal function with long-lasting results.

## Figures and Tables

**Table 1:** Summary of histological findings. ICRS II scores for patient repair tissue (italic) as well as healthy cartilage and marrow stimulation repair tissue for comparison. Some parameters could not be scored for the marrow stimulation repair tissue due to the loss of tissue architecture upon collection.

Histological parameter	Healthy cartilage biopsy score (positive control)	*Marrow stimulation + chondral allograft repair tissue biopsy score *	Marrow stimulation repair tissue biopsy score
Tissue morphology	100	*91 *	56.5

Percentage			
Hyaline cartilage	100	*85 *	5
Hyaline-like cartilage	0	*5 *	45
Fibrocartilage	0	*5 *	40
Fibrous connective tissue	0	*0 *	5
Bone	0	*0 *	0
Degenerative tissue	0	*5 *	5
Calcified cartilage	0	*0 *	0

Total percentage	100	*100 *	100

Matrix staining	90	*85 *	69.9
Cell morphology	96	*87.5 *	52.0
Chondrocyte clustering	100	*30 *	95
Surface architecture	90	*85 *	65
Basal integration	100	*90 *	n/a
Formation of a tidemark	100	*100 *	n/a
Subchondral bone abnormalities/marrow fibrosis	100	*95 *	n/a
Inflammation	100	*100 *	100
Abnormal calcification/ossification	100	*100 *	100
Vascularization	100	*100 *	80
Surface/superficial assessment	100	*65 *	n/a
Mid/deep zone assessment	100	*50 *	n/a
Overall assessment	98	*60 *	n/a
